# Antitumor activity of afatinib in EGFR T790M-negative human oral cancer therapeutically targets mTOR/Mcl-1 signaling axis

**DOI:** 10.1007/s13402-024-00962-6

**Published:** 2024-06-18

**Authors:** Jung-Min Han, Kyu-Young Oh, Su-Jung Choi, Won-Woo Lee, Bo-Hwan Jin, Ji-Hoon Kim, Hyun-Ju Yu, Ryan Jin Young Kim, Hye-Jung Yoon, Jae-Il Lee, Seong-Doo Hong, Sung-Dae Cho

**Affiliations:** 1 Department of Oral Pathology, School of Dentistry and Dental Research Institute, Seoul National University, Seoul, 03080 Republic of Korea; 2 Department of Oral Pathology, College of Dentistry, Dankook University, Cheonan, 31116 Republic of Korea; 3 Laboratory Animal Center, CHA University, CHA Biocomplex, Sampyeong-Dong, Seongnam, 13488 Republic of Korea; 4 Department of Dental Science, School of Dentistry and Dental Research Institute, Seoul National University, Seoul, 03080 Republic of Korea

**Keywords:** Head and neck cancer, Afatinib, Epidermal growth factor receptor, Myeloid cell leukemia-1, Mammalian target of rapamycin

## Abstract

**Purpose:**

This study investigates the role and effectiveness of the epidermal growth factor receptor (EGFR) tyrosine kinase inhibitor (TKI) in oral cancer, focusing on the clinical relevance of EGFR and myeloid cell leukemia-1 (Mcl-1) in head and neck cancers (HNCs). It aims to explore the molecular mechanism of afatinib, a TKI, in treating human oral cancer.

**Methods:**

We conducted an in *silico* analysis using databases like The Cancer Genome Atlas, Gene Expression Omnibus, and Clinical Proteomic Tumor Analysis Consortium, along with immunohistochemistry staining, to study EGFR and Mcl-1 expression in HNCs. For investigating afatinib’s anticancer properties, we performed various in vitro and in vivo analyses, including trypan blue exclusion assay, Western blotting, 4′-6-diamidino-2-phenylindole staining, flow cytometry, quantitative real-time PCR, Mitochondrial membrane potential assay, overexpression vector construction, transient transfection, and a tumor xenograft model.

**Results:**

Higher expression levels of EGFR and Mcl-1 were observed in HNC patient tissues compared to normal tissues, with their co-expression significantly linked to poor prognosis. There was a strong correlation between EGFR and Mcl-1 expressions in oral cancer patients. Afatinib treatment induced apoptosis and suppressed Mcl-1 in oral cancer cell lines without the EGFR T790M mutation. The mechanism of afatinib-induced apoptosis involved the EGFR/mTOR/Mcl-1 axis, as shown by the effects of mTOR activator MHY1485 and inhibitor rapamycin. Afatinib also increased Bim expression, mitochondrial membrane permeabilization, and cytochrome c release. It significantly lowered tumor volume without affecting body, liver, and kidney weights.

**Conclusion:**

Afatinib, targeting the EGFR/mTOR/Mcl-1 axis, shows promise as a therapeutic strategy for oral cancer, especially in patients with high EGFR and Mcl-1 expressions.

**Supplementary Information:**

The online version contains supplementary material available at 10.1007/s13402-024-00962-6.

## Introduction

Epidermal growth factor receptor (EGFR), which is a transmembrane receptor tyrosine kinase (RTK) with a molecular weight of 170 kDa and is a member of the human epidermal growth factor receptor (HER) family, plays a key role in the maintenance of epithelial tissue [1]. During organogenesis and tissue regeneration, the EGFR normally provides a robust signal for epithelial cell growth and survival by binding of ligands on the cell surface, leading to homo- or hetero-dimerization and inducing its activation [2, 3]. Pathologically, EGFR’s role in the development of human cancers through its dysregulation, including its overexpression, overproduction of its ligands, and altered triggering of its tyrosine kinase domain (TKD) activity by point mutation, has been well studied [4, 5]. The majority of solid tumors including lung, pancreatic, and prostate cancers and gliomas are overexpressed in EGFR [6–9]. Especially, EGFR is more highly overexpressed (80–90%) in head and neck cancers (HNCs) than in any other cancer types, and this overexpression is strongly associated with a poor prognosis of HNC patients [10], suggesting that EGFR-targeted therapy may be a significant interest in HNC.

EGFR is commonly targeted either by monoclonal antibodies (mAbs) or small molecule tyrosine kinase inhibitors (TKIs). mAbs target extracellular domain of EGFR and block the endogenous ligands from binding to EGFR [11]. Cetuximab is the only EGFR mAb, targeting the RTK pathway that is FDA approved for the treatment of HNC in 2006 [12]. It was also used for advanced HNC in combination with radiotherapy or platinum-based chemotherapy (PBC) after failure of PBC [13, 14]. However, Xiang et al. [15] recently reported that cetuximab treatment was associated with a significantly higher cancer-specific mortality than cisplatin treatment, suggesting that cetuximab may be a less preferred chemotherapeutic agent for HNC. Small-molecule TKIs diffuse into cells and bind to TK of the EGFR. Gefitinib (a reverse-binding TKI) and afatinib (an irreversible-binding TKI) were approved as lung cancer treatments in 2003 and 2013, respectively [16]. However, there has been no FDA approval of EGFR-TKIs for HNC to date. Additionally, although the anticancer activity and associated mechanisms of EGFR-TKIs in other cancers have been well understood, supportive preclinical studies for the clinical potential of EGFR-TKIs in HNC are lacking. Therefore, in the present study, we aimed to investigate the clinical significance of EGFR-TKIs as an anticancer drug candidate and to elucidate the molecular mechanism underlying its anticancer activity in human oral cancer.

## Results

### High co-expressions of EGFR and myeloid cell leukemia-1 (Mcl-1) are associated with a poor prognosis among patients with oral cancer

**Fig. 1:**
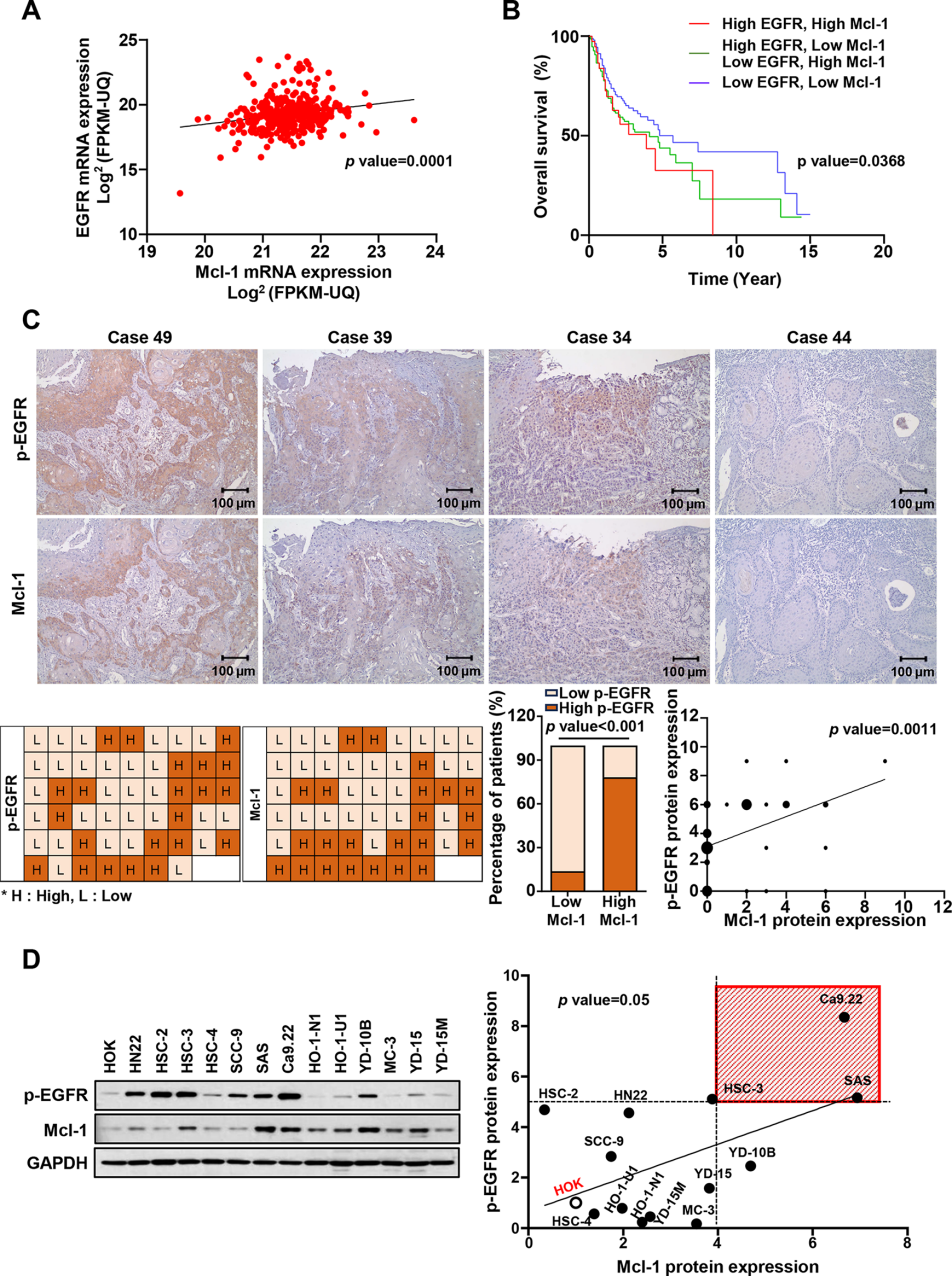
The correlation between EGFR and Mcl-1 expressions in HNC and oral cancer patients. **A**. Analysis of the correlation between EGFR and Mcl-1 mRNA expressions in HNC patients from the TCGA dataset. **B**. KM plotter analysis of the association between concurrent expression of EGFR and Mcl-1 and survival rate in HNC patients. **C**. IHC analysis of the association between the protein expression levels of p-EGFR and Mcl-1 in the tissues of 52 oral cancer patients (**upper panel**), The representative images of p-EGFR and Mcl-1 expression (cases 49, 39, and 34 for pEGFR+/Mcl-1 + and case 44 for pEGFR-/Mcl-1-) (**lower panel**) the graphical pattern of the corresponding IHC scores. **D**. Selection of oral cancer cell lines with high coexpression of p-EGFR and Mcl-1 (indicated by red box) using Western blotting in HOK and 13 oral cancer cell lines

To investigate the clinical significance of EGFR and Mcl-1 in human HNC pathogenesis, we performed an *in silico* analysis to evaluate the expression of EGFR and Mcl-1 in human HNC. EGFR and Mcl-1 mRNA and protein expression levels were significantly higher in HNC tissues than in normal or margin tissues using datasets obtained from the Gene Expression Omnibus (GEO), the Cancer Genome Atlas (TCGA), and Clinical Proteomic Tumor Analysis Consortium (CPTAC) (Additional File 1A–E, G–K). Using the KM plotter, we found a significant correlation between EGFR or Mcl-1 expressions and overall survival (OS) in patients with HNC. (Additional File 1 F and L). To investigate the correlation between the expression levels of EGFR and Mcl-1 in HNC, the TCGA database was used, and the expression of both mRNAs was in direct proportion to each other among HNC patients (Fig. 1A). The double high EGFR and Mcl-1 subgroup was also associated with a worse outcome in HNC patients as compared to the other subgroups (Fig. 1B). The results of immunohistochemical analysis unveiled that, among the 52 oral squamous cell carcinoma cases examined, 42.3% (22/52) and 44.2% (23/52) showed high expressions of p-EGFR and Mcl-1, respectively (Fig. 1C). Notably, Mcl-1 and p-EGFR exhibited a similar spatial distribution within the same tissue section in pEGFR+/Mcl-1 + cases (Fig. 1C). The expression level of Mcl-1 was found to be robustly correlated with that of p-EGFR (*p* < 0.001) (Fig. 1C). To investigate the correlation between the expressions of p-EGFR and Mcl-1 proteins in oral cancer cell lines, their expression levels were analyzed by western blotting in 13 oral cancer cell lines and human oral keratinocyte (HOK). The analysis revealed a correlation between the expression of p-EGFR and Mcl-1 proteins in oral cancer cell lines, with the highest coexpression observed in the OSCC cell lines HSC-3, SAS, and Ca9.22 (Fig. 1D). Taken together, these results indicate that EGFR and Mcl-1 are correlated and coexpressed in oral cancer, suggesting that both factors may contribute to poor prognosis among oral cancer patients.

### Afatinib shows superior anticancer activity and Mcl-1 suppression as compared to gefitinib in EGFR T790M-negative oral cancer cell lines

**Fig. 2:**
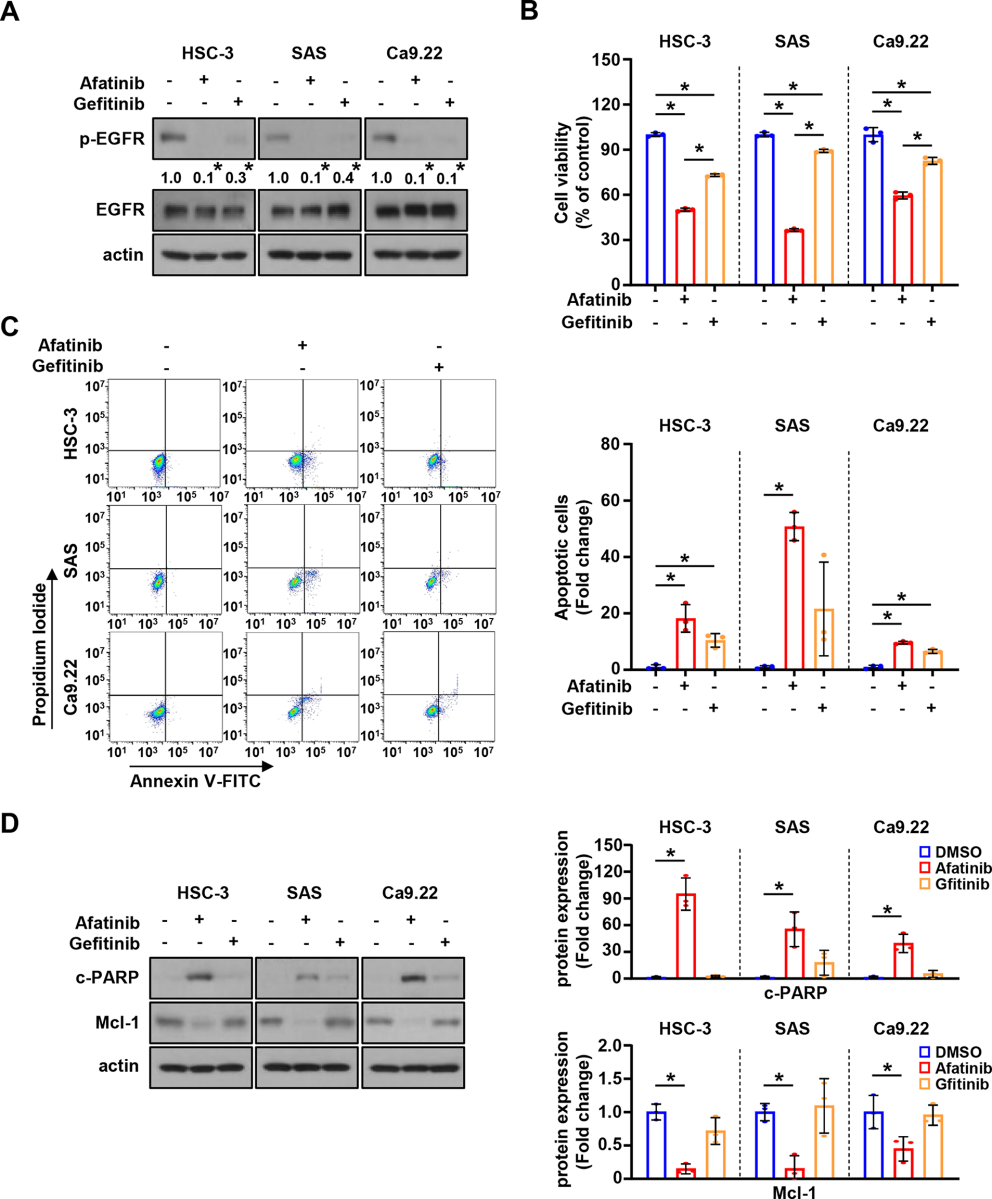
Comparison of the anticancer effects of first- (gefitinib) and second-generation EGFR-TKI (afatinib) in T790M mutation-negative oral cancer cell lines. **A**. Western blot analysis of p-EGFR and EGFR protein expressions in oral cancer cell lines treated with either gefitinib (8 µM) or afatinib (8 µM) for 24 h. Actin was used as the loading control. **B**. Trypan blue exclusion assay for cell viability. **C**. Annexin V/PI double staining to quantify the proportion of apoptotic cells. **D**. Western blot analysis of c-PARP and Mcl-1 protein expressions. All graphs are expressed as the mean ± SD of three independent experiments. *p < 0.05 by one-way ANOVA

To detect the T790M point mutation of EGFR in three selected cell lines, one-click Sanger sequencing was performed, and the results showed no T790M mutation in these cell lines (Additional File 2). As three cell lines are T790M-negative, we evaluated the potential anticancer effect of gefitinib (first-generation EGFR-TKI) and afatinib (second-generation EGFR-TKI). Although both gefitinib and afatinib appeared to inactivate EGFR, afatinib more dramatically inhibited cell growth as compared to gefitinib (Fig. 2A and B). Afatinib also induced a higher proportion of Annexin V-positive cells (Fig. 2C) and a higher expression of cleaved PARP than gefitinib, which was associated with decreased Mcl-1 expression (Fig. 2D). These results suggest that the second-generation EGFR-TKI, afatinib, has greater anticancer activity in OSCC cell lines as compared to the first-generation EGFR-TKI, gefitinib.

### Mcl-1 protein is closely involved in afatinib-induced apoptosis in oral cancer cell lines

**Fig. 3:**
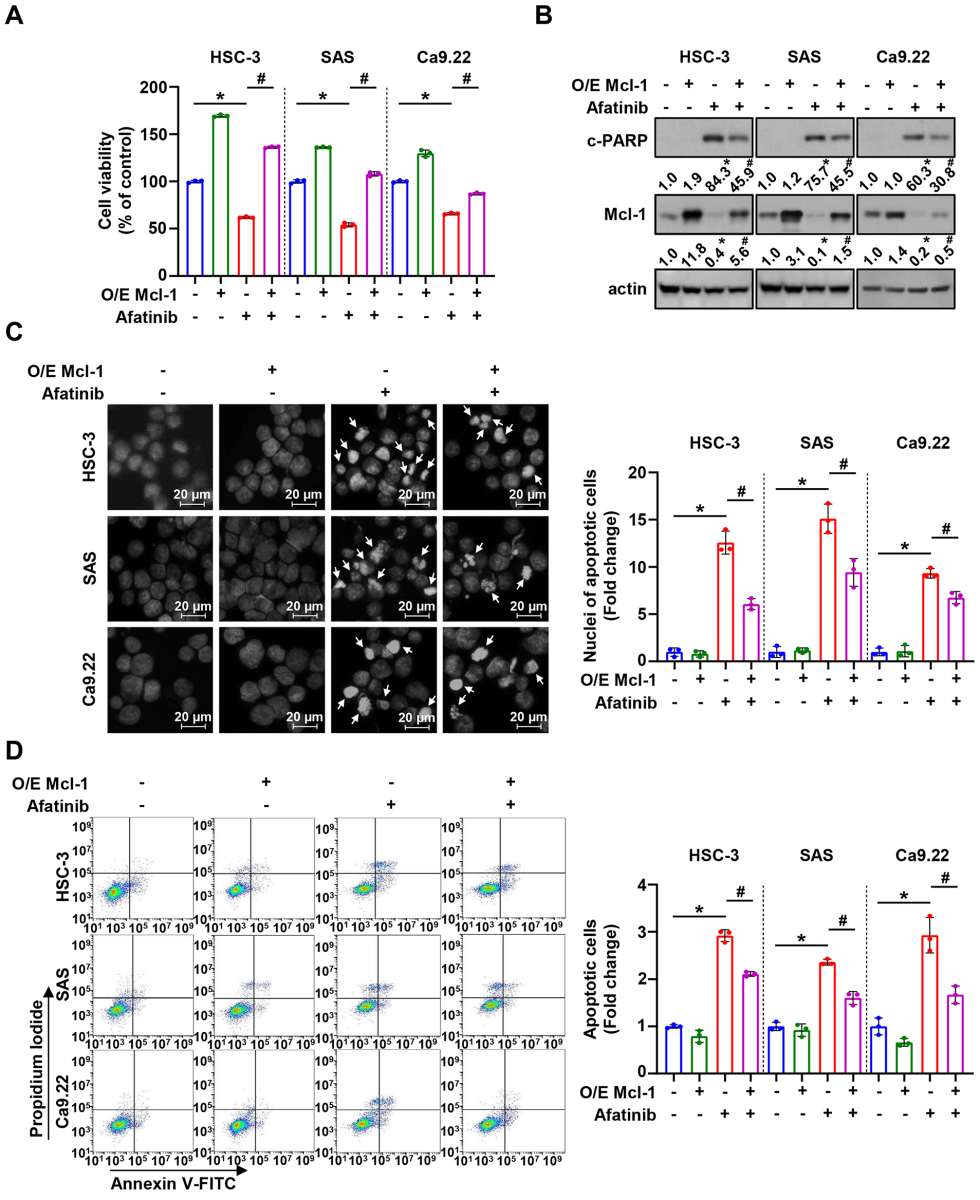
The role of Mcl-1 in afatinib-induced apoptosis. **A**. Measument of cell viability in afatinib (8 µM) -treated oral cancer cell lines with or without the Mcl-1 overexpression vector. **B**. Western blot analysis of c-PARP and Mcl-1 protein expressions. Actin was used as the loading control. DAPI staining (**C**) and Annexin V/PI double staining (**D**) to quantify the proportion of apoptotic cells. All graphs are expressed as the mean ± SD of three independent experiments. *p < 0.05 (compared with the control-treated group) and ^#^<0.05 (compared with afatinib-treated group) by one-way ANOVA

To determine whether afatinib-mediated apoptosis relies on Mcl-1, three cell lines were transfected with either an empty or Mcl-1 expressing vector. The overexpression of Mcl-1 significantly rescued cell viability and reversed afatinib-induced cleaved PARP in HSC-3, SAS, and Ca9.22 cell lines (Fig. 3A and B). These findings were further supported by the results demonstrating that the proportion of apoptotic nuclei was lower in Mcl-1 overexpressing cells than in the control group (Fig. 3C). Furthermore, the percentages of Annexin V-positive cells in Mcl-1 overexpressing cells were reduced in comparison to that of the control (Fig. 3D). These findings suggest that Mcl-1 may be the key molecule underlying afatinib-induced apoptosis in oral cancer cell lines.

### mTOR signaling is involved in afatinib-induced downregulation of Mcl-1 in oral cancer cell lines

**Fig. 4:**
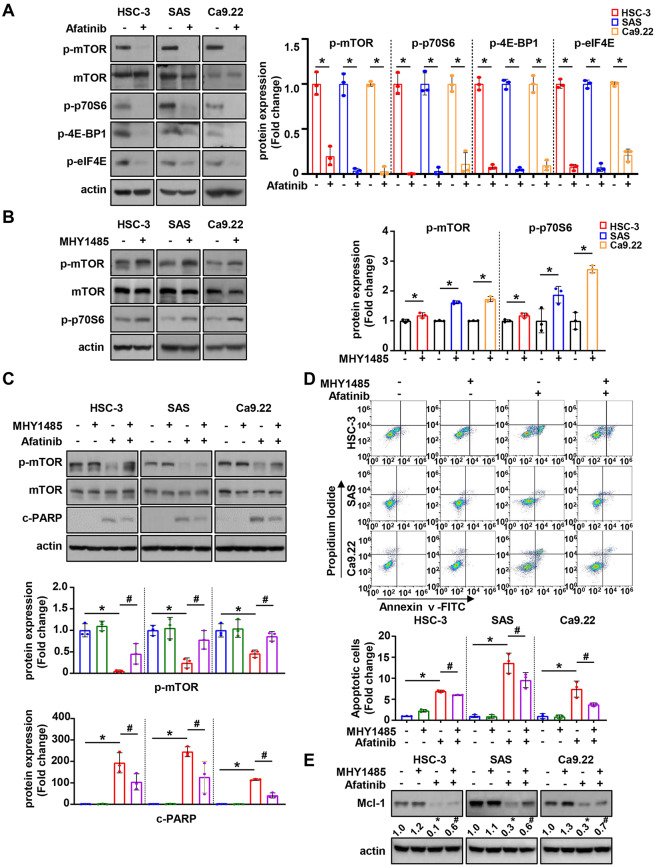
The involvement of mTOR signaling pathway in afatinib-induced apoptosis using MHY1485 (mTOR activator). **A**. Western blot analysis of p-mTOR, mTOR and p-p70S6 protein expressions in oral cancer cell lines treated with 8 µM of afatinib for 24 h. Actin was used as the loading control. **B**. Western blot analysis of p-mTOR, mTOR, and p-p70S6 protein expressions in oral cancer cell lines treated with MHY1485. **C**. Western blot analysis of p-mTOR, mTOR, and c-PARP expressions in oral cancer cell lines pretreated with MHY1485 for 1 h, followed by afatinib treatment. **D**. Annexin V/PI double staining to quantify the proportion of apoptotic cells. **E**. Western blot analysis of Mcl-1 expression in oral cancer cell lines pretreated with MHY1485, followed by afatinib treatment. All graphs are expressed as the mean ± SD of three independent experiments. *p < 0.05 by two-tailed Student’s t-test or one-way ANOVA. ^#^<0.05 (compared with afatinib-treated group) by one-way ANOVA

**Fig. 5:**
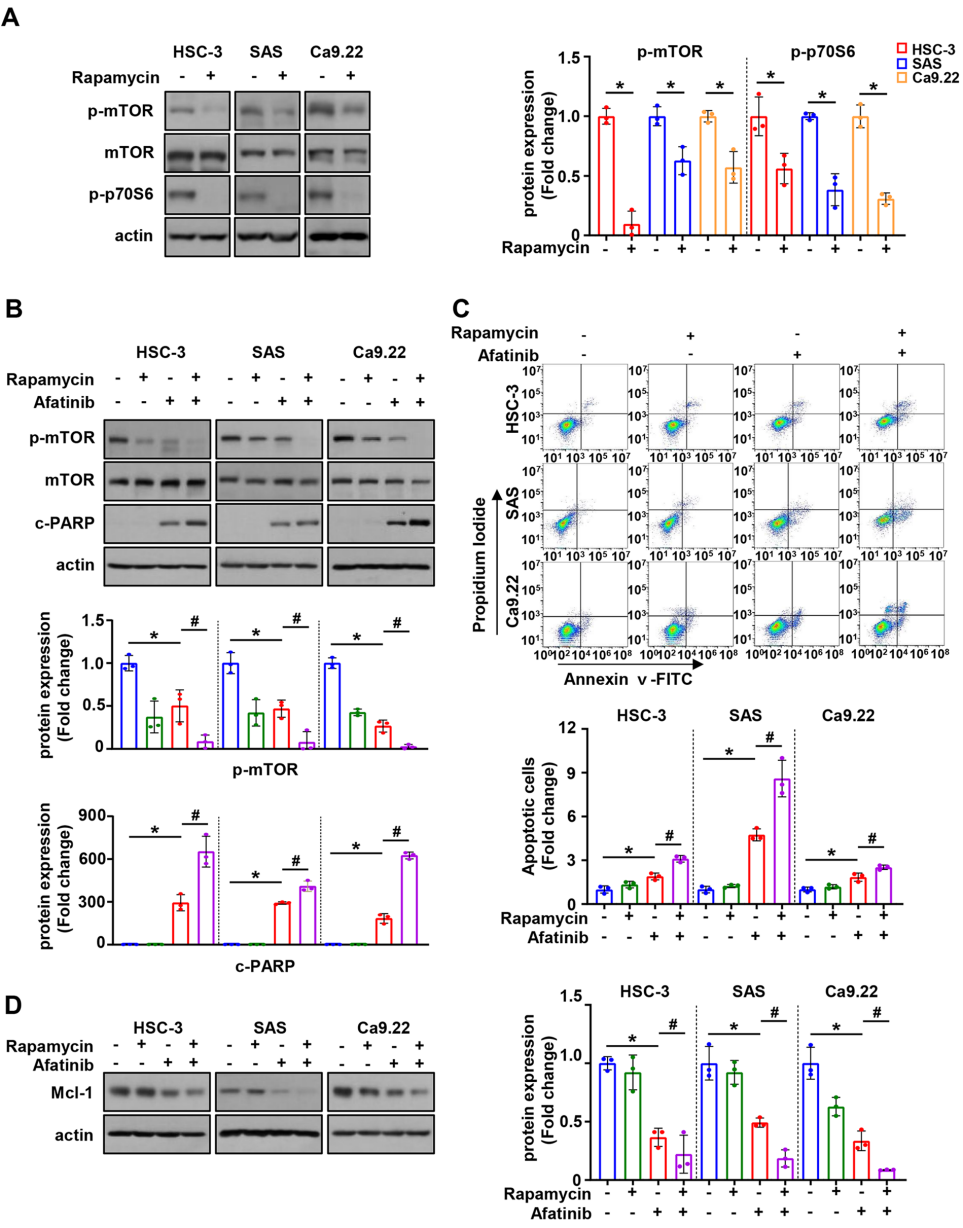
The involvement of mTOR signaling pathway in afatinib-induced apoptosis using rapamycin (mTOR inhibitor). **A**. Western blot analysis of p-mTOR, mTOR, and p-p70S6 protein expressions in oral cancer cell lines treated with rapamycin. Actin was used as the loading control. **B**. Western blot analysis of p-mTOR, mTOR, and c-PARP expressions in oral cancer cell lines pretreated with rapamycin for 1 h, followed by afatinib treatment (8 µM). **C**. Annexin V/PI double staining to quantify the proportion of apoptotic cells. **D**. Western blot analysis of Mcl-1 expression in oral cancer cell lines pretreated with rapamycin, followed by afatinib treatment. All graphs are expressed as the mean ± SD of three independent experiments. *p < 0.05 by two-tailed Student’s t-test or one-way ANOVA. ^#^<0.05 (compared with afatinib-treated group) by one-way ANOVA

Next, we investigated mTOR and its related proteins to determine the link between EGFR and Mcl-1 signaling by afatinib in oral cancer cell lines. Afatinib significantly reduced p-mTOR and p-P70S6K expressions (Fig. 4A). To determine whether the Mcl-1 protein reduction and apoptotic activity induced by afatinib were an mTOR-dependent, we used MHY1485 (mTOR activator) and rapamycin (mTOR inhibitor). The expression of p-mTOR and p-P70S6K was significantly increased in all three cell lines after MHY1485 treatment (Fig. 4B), whereas the opposite effect was observed by rapamycin (Fig. 5A). The introduction of p-mTOR by MHY1485 in the HSC-3, SAS, and Ca9.22 cell lines not only led to a reversal of the PARP cleavage impacted by afatinib (Fig. 4C), but also resulted in a reduction in the proportion of Annexin V-positive cells as compared to that of the control (Fig. 4D). Additionally, the introduction of p-mTOR by MHY1485 restored the Mcl-1 protein expression, which had been reduced by afatinib (Fig. 4D). Contrarily, the combination of afatinib with rapamycin significantly reduced cell viability and increased cleaved PARP expression and proportion of Annexin V-positive cells, as compared to the treatment with afatinib alone (Fig. 5B and C). Furthermore, the cotreatment of afatinib with rapamycin resulted in a more significant reduction in Mcl-1 expression than the treatment of afatinib alone (Fig. 5D). These results indicate that mTOR is effectively inactivated by afatinib and p-mTOR may regulate Mcl-1.

### Afatinib induces mitochondria-mediated apoptosis by upregulating bim protein expression in oral cancer cell lines

**Fig. 6:**
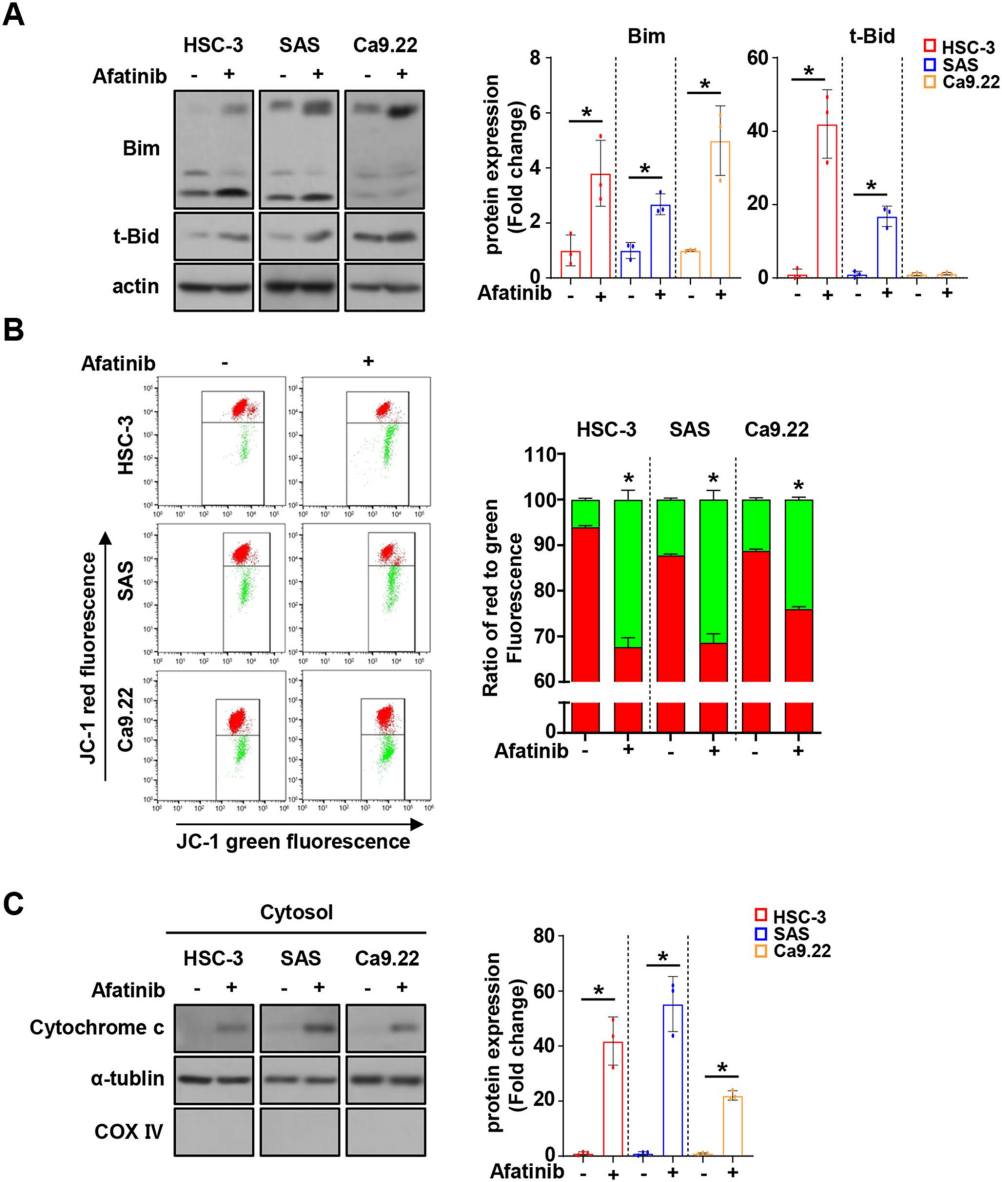
The effect of afatinib on mitochondria-dependent apoptosis in oral cancer cell lines. **A**. Western blot analysis of Bim and t-Bid protein expressions in oral cancer cell lines treated with 8 µM of afatinib for 24 h. Actin was used as the loading control. **B**. JC-1 assay for detecting MOMP (DΨm). **C**. Western blot analysis for the translocation of cytochrome c into cytosol. ɑ-Tubulin and COX IV were used as the loading control for the cytosol and mitochondria, respectively. All graphs are expressed as the mean ± SD of three independent experiments. *p < 0.05 by two-tailed Student’s t-test

The mitochondrial dependence of afatinib-mediated apoptosis was further investigated by analyzing the expression levels of BH3-only pro-apoptotic proteins (Bim and t-Bid). As shown in Fig. 6A, afatinib significantly increased Bim expression in all three cell lines, However, it appeared to affect t-Bid expression, but was not common to all three cell lines. To investigate whether afatinib induces apoptosis via mitochondrial dysfunction, JC-1 staining was performed. Afatinib treatment caused a significant reduction in red fluorescence as compared to the control group in all three cell lines, indicating that it induced the loss of ΔΨm (Fig. 6B). Additionally, afatinib significantly released cytochrome c into the cytosol (Fig. 6C). These results indicate that afatinib induces apoptosis in human OSCC cell lines via a mitochondrial dysfunction.

### Afatinib treatment in a xenograft mouse model shows significant reduction in tumor volume without detectable in vivo side effect

**Fig. 7:**
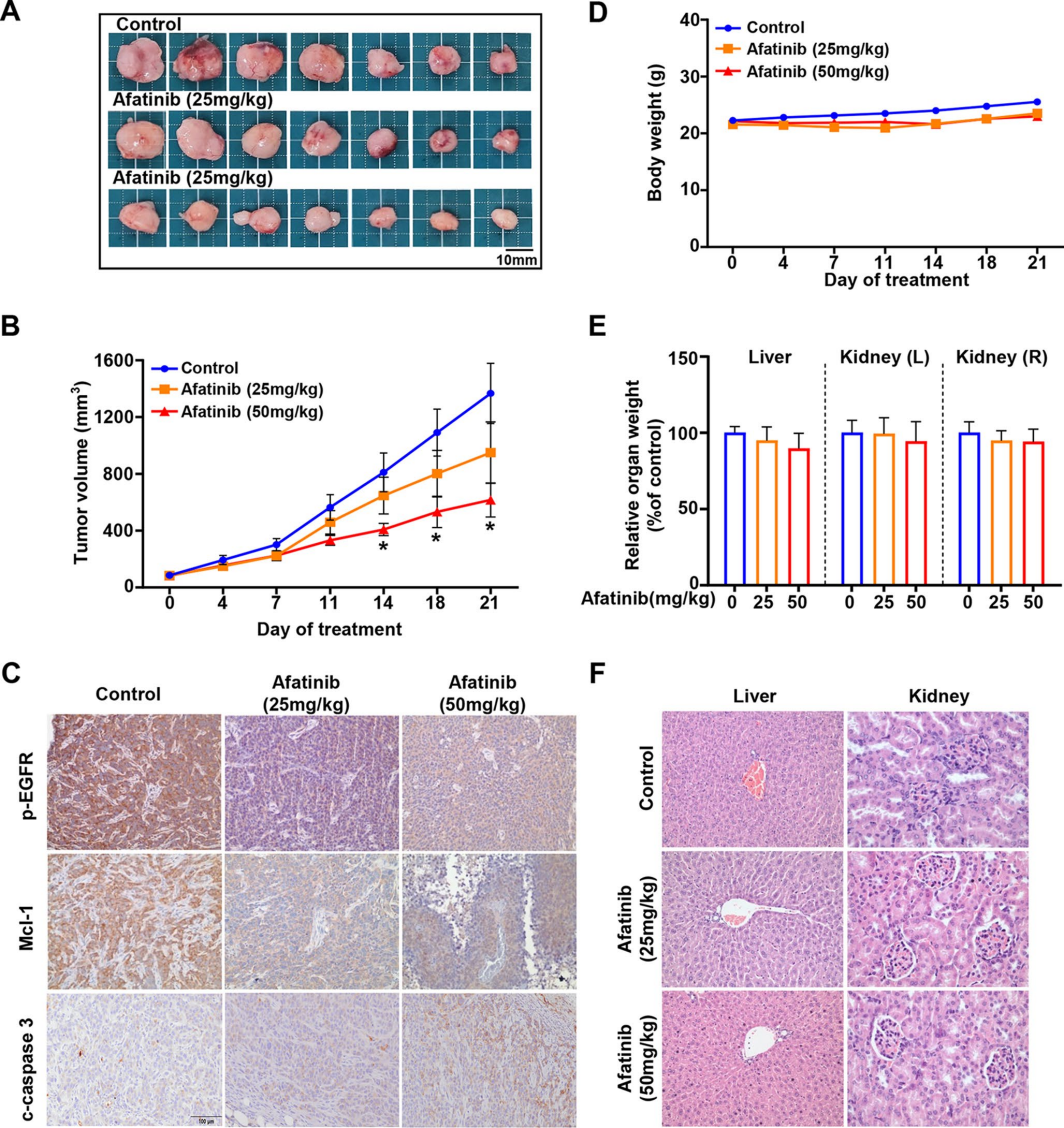
In vivo antitumor property of afatinib using a xenograft mouse model bearing HSC-3 cell line. Afatinib (25 and 50 mg/kg/day) was orally administered five times per week for 21 days to nude mice implanted with the HSC-3 cell line. **A**. Images of tumor specimens. **B**. Measurement of tumor volume. **C**. IHC analysis of tumor lysates for p-EGFR, Mcl-1, and c-caspase 3 (magnification, ×400). Measurement of body (**D**) and organ (liver and kidney) weights (**E**). **F**. H&E staining of tissues from control- and afatinib-treated mice. All graphs are expressed as the mean ± SD. *P < 0.05 by nonparametric Mann–Whitney tests

To evaluate the effectiveness of afatinib in inhibiting in vivo tumor growth, a xenograft mouse model was utilized by transplanting HSC-3 cells into the flank of athymic nude mice. Treatment with 50 mg/kg of afatinib led to a significant reduction in tumor volume on the 14th day after administration (Fig. 7A and B). The analysis of tumor lysates by IHC showed a decreased expression of p-EGFR and Mcl-1 and an increased expression of c-caspase 3 (Fig. 7C). Afatinib’s biocompatibility was assessed by monitoring the body weight and liver and kidney weights, revealing no significant changes following treatment (Fig. 7D and E). Additionally, histopathological findings showed that there were no differences in the two organs (liver and kidney) between the control- and afatinib-treated groups (Fig. 7F). These findings indicate that afatinib exhibits a potent antitumor activity without observable adverse effect in vivo.

## Discussion

Mutations in TKD of EGFR most commonly occur between exons 18 and 21. Alterations in exons 19 and 21 are most common in non-small cell lung cancer (NSCLC), explaining why this type of cancer is sensitive to EGFR-TKIs [17]. A retrospective study conducted in Saudi also showed that TKD, which spans exons 18 to 21, was mutated in approximately 57% of HNCs and its mutation was correlated with a higher grade of HNC, implying that EGFR mutations, which are known predictors of response to therapy in NSCLC, might be extrapolated to HNC [18]. Although EGFR-TKIs may be a promising treatment option for this reason, basic preclinical research of the efficacy of EGFR-TKIs in HNC is limited to date. In NSCLC, the most common EGFR mutations are exon 19 deletions and the L858R point mutation in exon 21 [19, 20]. Gefitinib (first-generation TKI) and afatinib (second-generation TKI) specifically targeted and inhibited these EGFR mutations [21]. It was found that T790M mutation is the common mechanism of resistance against gefitinib and afatinib [22] indicating that those two drugs may not be effective in EGFR T790M-positive cancers. In our one-click Sanger sequencing data, there was no T790M mutation in three oral cancer cell lines. That is why we chose gefitinib and afatinib to evaluate their potential anticancer effects in OSCC cell lines in the present study. Our work revealed that both EGFR-TKIs effectively inhibited p-EGFR, but afatinib-induced apoptosis to a much greater extent than gefitinib. This may be due to several reasons including (i) reversible vs. irreversible binding and (ii) EGFR-specific vs. Pan-HER-targeted. Several ongoing clinical trials are investigating the efficacy and safety of EGFR-TKIs in HNC. Gefitinib reportedly had no survival or outcome benefit in a clinical trial (phase III) in patients with metastatic or recurrent HNC [23]. Contrarily, afatinib showed an antitumor activity comparable to that cetuximab among patients with recurrent or metastatic HNC in clinical trial study (phase II). These suggest that irreversible pan-HER TKIs holds great potential to improve the treatment outcomes for patients with HNC.

Mcl-1, a member of the Bcl-2 family with antiapoptotic properties, promotes cell survival by inhibiting apoptosis [24]. Our group has previously shown that Mcl-1 is overexpressed in oral cancer and is closely associated with poor clinical outcomes, suggesting that the inhibition of Mcl-1 might be a good strategy for the treatment of oral cancer [25, 26]. Mcl-1 regulates mitochondrial outer membrane permeabilization, which is a vital process in the initiation of apoptosis, by engaging the interaction with BH3-only proteins, such as Bim, and t-Bid. Because our data showed afatinib induced loss of mitochondrial membrane potential to release cytochrome c from the mitochondria, the impact of afatinib on the expression patterns of Bim and t-Bid was assessed in our study. Our study findings revealed a notable upregulation in Bim expression. However, t-Bid expression was not commonly affected in all three cell lines. Bim activation is crucial for inducing apoptosis in EGFR-mutant NSCLC triggered by EGFR-TKIs [27, 28]. A previous meta-analysis also demonstrated that NSCLC patients harboring a deletion polymorphism in the Bim gene exhibited a diminished response to EGFR-TKIs [29] indicating that Bim may be essential for deriving therapeutic benefits from afatinib treatment in HNC.

Mcl-1 is known to be regulated by EGFR signaling pathway in cancer [30, 31], but there is a lack of research into the clinical correlation between EGFR and Mcl-1 in cancer, particularly, oral cancer. In the present study, we performed an *in silico* analysis and IHC using clinical samples. Booy et al. [32] reported that increased expression of EGFR is associated with a higher median Mcl-1 H-score using a breast tumor tissue microarray, supporting our present findings. Additionally, EGFR signaling reportedly regulated Mcl-1 survival action in neuroblastoma by disrupting Bim to Mcl-1 [33]. These suggest that Mcl-1 is a critical downstream molecule involved in the survival benefits conferred by activating the EGFR signaling pathway. Given the close relationship between EGFR and Mcl-1, the combination therapy of an EGFR inhibitor and a Mcl-1 inhibitor is worthy of evaluation for future direction. The activation of EGFR initiates a cascade of downstream signaling pathways that regulate various cellular processes, including cell growth, survival, proliferation, and differentiation. The major downstream signaling pathways activated by EGFR include the Ras/Raft/MEK/ERK, PI3K/Akt/mTOR, and STAT pathways. Given the close relationship between EGFR and Mcl-1 regulation, it is hypothesized that downstream signaling pathways of EGFR may play a role in regulating Mcl-1. In this study, additional experiments were conducted to elucidate the regulatory mechanism of Mcl-1. An intriguing finding form the experiment was that afatinib did not exert a significant impact on its mRNA or protein stability (data not shown), suggesting that afatinib may regulate Mcl-1 in a translation-dependent manner. Among the downstream signaling of EGFR, Mcl-1 synthesis involves of cap-dependent translation control, which is positively regulated by mTOR [34]. Thus, we conducted further investigations to verify the participation of mTOR signaling pathway in afatinib-mediated regulation of Mcl-1. The experimental results provided evidence for mTOR involvement in translational modification of Mcl-1 by afatinib. Kim et al. [35] previously reported that the combination of an inhibitor of glucose metabolism and afatinib in NSCLC resulted in a selective decrease in Mcl-1 expression through a translational control, mediated by the alteration of AMPK/mTOR signaling pathway. Our findings were consistent with those observed in NSCLC. To the best of our knowledge, the present study is the first to report that afatinib induces apoptosis through the EGFR/mTOR/Mcl-1 axis.

## Conclusions

Our study provides evidence that the concurrent expression of EGFR and Mcl-1 is linked to a poor prognosis in oral cancer patients. We demonstrate that afatinib, a second-generation EGFR-TKI, exhibits stronger anticancer effects compared to gefitinib in oral cancer cell lines lacking the EGFR790M mutation. Afatinib triggers mitochondria-dependent apoptosis by modulating the EGFR/mTOR/Mcl-1 signaling pathway. Our findings suggest that targeting the EGFR/mTOR/Mcl-1 axis with afatinib could be a promising therapeutic strategy for oral cancer treatment, particularly in patients with high EGFR and Mcl-1 co-expression.

## Methods

### The cancer genome atlas (TCGA) database

The HNC dataset from TCGA database (https://portal.gdc.cancer.gov/) was utilized to examine the correlation of mRNA expression between EGFR and Mcl-1 in HNC (*n* = 369). The process of trimming data was done using Jupyter Notebook and Pandas with Python 3.0, and the code is available at https://github.com/kunalchawlaa/TCGA-Oral-Cancer.

### Kaplan–Meier (KM) plotter survival analysis

Using the online survival analysis software KM plotter (http://kmplot.com/analysis), the OS rate of HNC patients was analyzed according to the expression levels of EGFR and Mcl-1.

### Clinical tissue samples

Between 2006 and 2007, the Department of Oral Maxillofacial Surgery at Seoul National University Dental Hospital in Seoul, Republic of Korea, collected 52 tissues from patients who had undergone surgical treatment for oral cancer. These tissues were then examined by immunohistochemistry (IHC). This retrospective study received approval from the Institutional Review Board (IRB) of Seoul National University Dental Hospital (IRB No. ERI20021).

### IHC staining

In the present study, paraffin-embedded tissues from oral cancer patients were sectioned at 4-µm thickness. The paraffin was melted by holding the sections at 60 °C for 1 h, followed by washing with neo-clear and graded ethanol to rehydrate the specimens. For antigen retrieval, the sections were microwaved in an antigen retrieval citrate buffer (pH 6.0) for 10 min, and then treated with a peroxidase blocking reagent (Dako, Carprinteria, CA, USA) for 5 min to inactivate the endogenous peroxidase activity. Sections were incubated overnight at 4 °C in a humidified chamber with a primary antibody against p-EGFR (Cat. No. 3777; 1:100) or Mcl-1 (Cat. No. 39,224; 1:100). The next day, the sections were reacted with REALTM EnVisionTM/horseradish peroxidase (HRP) Rabbit/Mouse (Dako) for 30 min at room temperature (RT), followed by a color reaction with REALTM DAB and Chromogen with Substrate Buffer (Dako) for 30 sc. The sections were then counterstained with hematoxylin, dehydrated, and mounted with a Permount solution (Thermo Fisher Scientific, Waltham, MA, USA).

### Evaluation of IHC staining results

Immunohistochemically stained sections were semi-quantitatively evaluated by two oral pathologists. Tumor cells showing cytoplasmic staining were regarded as positive cells. The intensity of staining (0, negative; 1, weak; 2, moderate; and 3, strong) and percentage of positive cells (0, 0%; 1, 1–9%; 2, 10–49%; 3, 50–100%) were scored. The final immunohistochemical scores were established by multiplying the intensity and percentage scores. The final scores of ≥ 6 and ≥ 2 were classified as high expression and scores of < 6 and < 2 as low expression for p-EGFR and Mcl-1, respectively.

### Cell culture and reagents

The HOK cell line was obtained from Lifeline Cell Technology (Oceanside, CA, USA). The HSC-2, HSC-3, HSC-4, SAS, and Ca9.22 cell lines were kindly provided by Hokkaido University (Hokkaido, Japan). The HN22 and MC-3 cell lines were given by Dankook University (Cheonan, Republic of Korea) and Fourth Military Medical University (Xi’an, China), respectively. The HO-1-N1 and HO-1-U1 cell lines were purchased from the Japanese Collection of Research Bioresources Cell Bank (Osaka, Japan). YD-10B, YD-15, and YD-15 M cell lines were obtained from the Korean Cell Line Bank (Seoul, Republic of Korea). The HOK cell line was grown using the DermaLife K Keratinocyte Medium Complete Kit (Lifeline Cell Technology, Frederick, MD, USA). The HN22, HSC-2, HSC-3, HSC-4, SCC-9, SAS, Ca9.22, HO-1-N1, HO-1-U1, and MC-3 cell lines were maintained in Dulbecco’s modified Eagle’s medium/nutrient mixture F-12 (WELGENE, Gyeongsan, Republic of Korea). The YD-10B, YD-15, and YD-15 M cell lines were cultured in Roswell Park Memorial Institute (RPMI) 1640 medium (WELGENE). The culture media used for all cell lines were supplemented with 10% fetal bovine serum (WELGENE) and 1% penicillin/streptomycin (WELGENE), and the cells were maintained at 37 ºC in a humidified atmosphere with 5% CO_2_. The cells were rinsed with Dulbecco’s phosphate-buffered saline. Afatinib (BIBW2992), gefitinib (ZD1839), and MHY 1485 were purchased from Selleck Chemicals (Houston, TX, USA), and rapamycin was obtained from Sigma-Aldrich (St. Louis, MA, USA). The chemical compounds were dissolved in dimethyl sulfoxide and aliquoted, then stored at − 20 °C.

### Western blotting

A 1× RIPA lysis buffer (Millipore Corp, Burlington, MA, USA) in combination with phosphatase inhibitor tablets (Thermo Scientific Inc., Rockford, IL, USA) and protease inhibitor cocktails (Roche, Mannheim, Germany) was used to extract total protein from human oral cancer cell lines. Each sample’s protein concentration was determined using a DC Protein Assay Kit (BIO-RAD Laboratories, Madison, WI, USA). An equal amount of protein from each sample was then heated with 5× protein sample buffer at 95 °C for 10 min, separated by SDS-PAGE, and transferred to the immunoblot PVDF membranes (Pall Life Sciences, Portsmouth, Hampshire, England). After blocking with 5% skim milk in Tris-buffered saline with Tween 20 for 1.5 h at RT, the membranes were incubated overnight with the specified primary antibodies at 4 °C. After washing, the membranes were incubated for 2 h at RT with the appropriate secondary antibodies conjugated to HRP. Immunoreactive protein bands were detected using either an x-ray film or the Image Quant LAS 500 system (GE Healthcare Life Sciences, Piscataway, NJ, USA) with a WestGlow™ FEMTO chemiluminescent substrate (BIOMAX, Seoul, Republic of Korea). ImageJ software was used to calculate the protein levels. The following primary antibodies were used in the experiments: rabbit antihuman polyclonal antibodies against cleaved PARP (Cat. No. 9541; 1:1000), cleaved caspase-3 (Cat. No. 9664; 1:1000), phospho-mTOR (Cat. No. 2971; 1:1000), phospho-p70S6 (Cat. No. 9205; 1:1000), Bim (Cat. No. 2819; 1:1000), Mcl-1 (Cat. No. 4572; 1:1000), and rabbit antihuman monoclonal antibodies against phospho-EGFR (Cat. No. 3777; 1:1000), mTOR (Cat. No. 2983; 1:1000), and c-MYC (Cat. No. 5605; 1:1000) that were purchased from Cell Signaling Technology, Inc (Danvers, MA, USA); and rabbit antihuman polyclonal antibody against EGFR (Cat. No. sc-03; 1:1000), goat antihuman polyclonal antibody against t-Bid (Cat. No. 34,325; 1:1000), and mouse antihuman monoclonal antibodies against β-actin (Cat. No. 47,778; 1:3000) and GAPDH (Cat. No. ab9484; 1:3000) that were purchased from Santa Cruz Biotechnology, Inc (Santa Cruz, CA, USA).

### Trypan blue exclusion assay

Cells were dissociated from the culture plate using a 0.25% trypsin-EDTA solution (WELGENE), and then resuspended in 1 mL of PBS. A 0.4% trypan blue (Gibco, Paisley, UK) solution was applied to the cells for staining, and a CytoSMART automatic cell counter (Corning, Tewksbury, MA, USA) was used to determine the number of viable cells in the sample. Only trypan blue-unstained cells were counted as viable.

### Annexin V/propidium iodide (PI) staining

The presence of apoptosis was assessed using the FITC Annexin V apoptosis detection kit (BD Pharmingen, San Jose, CA, USA). The harvested cells were rinsed twice with PBS and then exposed to Annexin V-FITC and PI dyes for 15 min at RT. The stained cells were then examined using a FACS Caliber instrument, and the resulting measurements were calculated using Cell Quest software (BD Biosciences).

### 4′-6-Diamidino-2-phenylindole (DAPI) staining

A DAPI solution (Sigma-Aldrich) was used to examine the changes in the nuclear morphology of the apoptotic cells. Cells were seeded onto 60 mm^2^ plates and treated with 8 µM of afatinib for 24 h. Following the treatment, the cells were collected, washed twice with PBS, and then fixed with 100% methanol RT for 10 min. The cells were then washed again with PBS, seeded onto glass slides coated with a layer of the substance, and stained with a DAPI solution (2 µg/ml). The cell morphology changes were visualized and analyzed using a fluorescence microscope.

### Quantitative real time PCR (qPCR)

The level of Mcl-1 mRNA was quantified through qPCR analysis. After obtaining the target cDNA, PCR was performed using AMPIGENE qPCR Green Mix Hi-Rox (Enzo Life Sciences, Inc, Farmingdale, NY, USA). The qPCR was carried out using the StepOne Plus Real-Time PCR System. The qPCR was carried out using the StepOne Plus Real-Time PCR System (Applied Biosystems, Foster city, CA, USA) and amplification of the target cDNA was achieved using the primers listed below: sense 5’-GTA TCA CAG ACG TTC TCG TAA GG-3’, antisense 5’-CCA CCT TCT AGG TCC TCT ACA T-3’ for MCL-1 and sense 5’- GTG GTC TCC TCT GAC TTC AAC-3’, antisense 5’- CCT GTT GCT GTA GCC AAA TTC-3’ for GAPDH. The MCL-1 and GAPDH amplification was carried out over a total of 40 cycles (for 2 min at 95℃, for 10 s at 95℃, and for 30 s at 60℃). PCR amplification was performed in triplicate for each sample, and the relative expression of Mcl-1 mRNA was determined using the 2-ΔΔCt.

### Construction of Mcl-1 overexpression vector and transient transfection

An open reading frame of the human Mcl-1 gene (NM_021960) was obtained by cDNA amplification using a pair of primers. The primer sequences were as follows: Mcl-1 sense 5′-GAA TTC ATG TTT GGC CTC AAA AGA‐3′ (containing an EcoRI site) and Mcl-1 antisense 5′‐GAA TTC CTA TCT TAT TAG ATA TGC‐3′ (containing an EcoRI site). The PCR product was then ligated into the pGEM®‐T Easy Vector System (Promega, Madison, WI, USA) for cloning. The target genes of interest were successfully inserted into the multiple cloning site of the pcDNA3.1 (+) vector (Invitrogen, Carlsbad, CA, USA). The HSC-3, SAS, and Ca9.22 cell lines were used for the transfection experiments. Cells were transfected with either an empty pcDNA3.1 vector or a pcDNA3.1-Mcl-1 vector construct (0.5 µg) using Lipofectamine 2000 Reagent (Invitrogen) as the transfection agent, according to the manufacturer’s instructions.

### Mitochondrial membrane potential (ΔΨm) assay

The change in ΔΨm was evaluated using a MitoScreen assay kit (BD Pharmingen). After harvesting, the cells were rinsed twice with PBS and then exposed to a JC-1 staining solution for 15 min at 37 °C. The cells were rinsed twice with an 1 × assay buffer, and the JC-1 fluorescence was measured by flow cytometry.

### Nude mouse xenograft assay

Six-week-old male BALB/c-nude mice were obtained from JA BIO, Inc (Suwon, Republic of Korea). The mice used in the study were treated according to the CHA University Institutional Animal Care and Use Committee (IACUC) guidelines (IACUC approval number: 230,040). A fixed number of HSC-3 cells were transplanted subcutaneously into the flanks of the mice. For the tumor-bearing mice, the vehicle control and afatinib (25 and 50 mg/kg/day) were administered orally by gavage five times a week for 21 days starting at approximately 7 days after the start of the experiment (day 0). Tumor volume and body weight of the mice were measured twice a week, and the tumor weight was measured on the day of necropsy. The tumor volume was determined by measuring its diameter along two axes using calipers, and then calculating the volume using the formula V = π/6[(D + d)/2], where D and d are the larger and smaller diameters, respectively.

### Statistical analysis

For *in silico* studies, a paired or unpaired two-tailed Student’s t-test was used to compare the EGFR and Mcl-1 expression levels between the normal and tumor tissues. Spearman’s rank correlation analysis was used to examine the relationship between the DNA copy number and mRNA expression levels. In vitro studies utilized a two-tailed Student’s t-test to assess the significance of the differences between two experimental groups, and a one-way analysis of variance was performed for multiple comparisons using Tukey’s post hoc test. Nonparametric Mann–Whitney tests were used to analyze non-normally distributed datasets in the in vivo studies. The association between the immunohistochemical expression of Mcl-1 and p-EGFR in oral cancer tissue samples was analyzed using Pearson’s chi-square test. Data analysis was performed with GraphPad Prism version 8.4 and evaluated with SPSS 25 (SPSS, Chicago, IL, USA). All experiments were conducted independently in triplicate. The results with *P* < 0.05 were considered statistically significant.

## Electronic supplementary material

Below is the link to the electronic supplementary material.

Supplementary Material 1

## Data Availability

All publicly accessed data are available on databases described in methodology. The datasets analyzed during the current study are available from the corresponding author on reasonable request.
