# Supplementary material for: Antitumor activity of afatinib in EGFR T790M-negative human oral cancer therapeutically targets mTOR/Mcl-1 signaling axis
Source: Cell Oncol (Dordr). 2024 Jun 18;48(1):123–38. doi: 10.1007/s13402-024-00962-6 (PMC11850456; doi:10.1007/s13402-024-00962-6)
Supplement: Supplementary file 1 — Supplementary Material 1 [file 13402_2024_962_MOESM1_ESM.docx]

**Supplementary materials and methods**

***Gene Expression Omnibus (GEO) database***

The public genomics database, GEO (https://www.ncbi.nlm.nih.gov/geo/), was used to explore the mRNA expression levels of EGFR and Mcl-1 in HNC. The differences in EGFR (1956_at) mRNA levels were analyzed among the normal (n = 24), margin (n = 49), and cancer (n=23) groups using the GEO series GSE31056. The GEO series GSE30784 reporter identifier 201983_s_at was utilized to assess the differences in EGFR mRNA levels among the normal (n = 45), dysplasia (n = 17), and cancer (n = 167) groups. The variation in EGFR mRNA levels was examined within the same cases, comparing adjacent non-tumor epithelium (n = 40) and cancer cells (n = 40) using the GEO series GSE37991 reporter identifier ILMN_1755535. Additionally, the mRNA levels of Mcl-1 were assessed in the normal (n = 45), dysplasia (n = 17), and cancer (n = 167) groups using the GEO series GSE30784 with reporter identifiers 200796_s_at, 200797_s_at, 200798_x_at, and 214056_at, to determine the variation in expression between these groups. To ensure consistency and accuracy in the analysis, all the extracted data underwent normalization using Geo2R, a tool provided by the GED database.

***Clinical Proteomic Tumor Analysis Consortium (CPTAC) database***

The CPTAC database (https://pdc.cancer.gov/pdc/) was used to estimate the results of the proteomic analyses. Reporter ion intensity log2 ratio unshared peptides of EGFR and Mcl-1 values are shown to compare the expression levels of EGFR and Mcl-1 proteins between the normal tissue (n = 33) and tumor samples (n = 57).

***EGFR mutation assay***

The genomic DNA from oral cancer cell lines was isolated using the InstaGene Matrix (Bio-Rad Laboratories, Hercules, CA, USA) method. EGFR exon 20 was amplified with the following primers: sense 5’-GTA AAA CGA CGG CCA GT-3’ and antisense 5’-GCG GAT AAC AAT TTC ACA CAG G-3’. PCR amplification was carried out over a total of 35 cycles (for 30 seconds at 95 ºC, 30 seconds at 58 ºC, and 1 minute at 72 ºC). The PCR products were purified using ExoSAP-IT (Thermo Fisher Scientific, Waltham, MA, USA), and the samples were Sanger sequenced using the Applied Biosystems ABI PRISM 3730XL Analyzer.

**
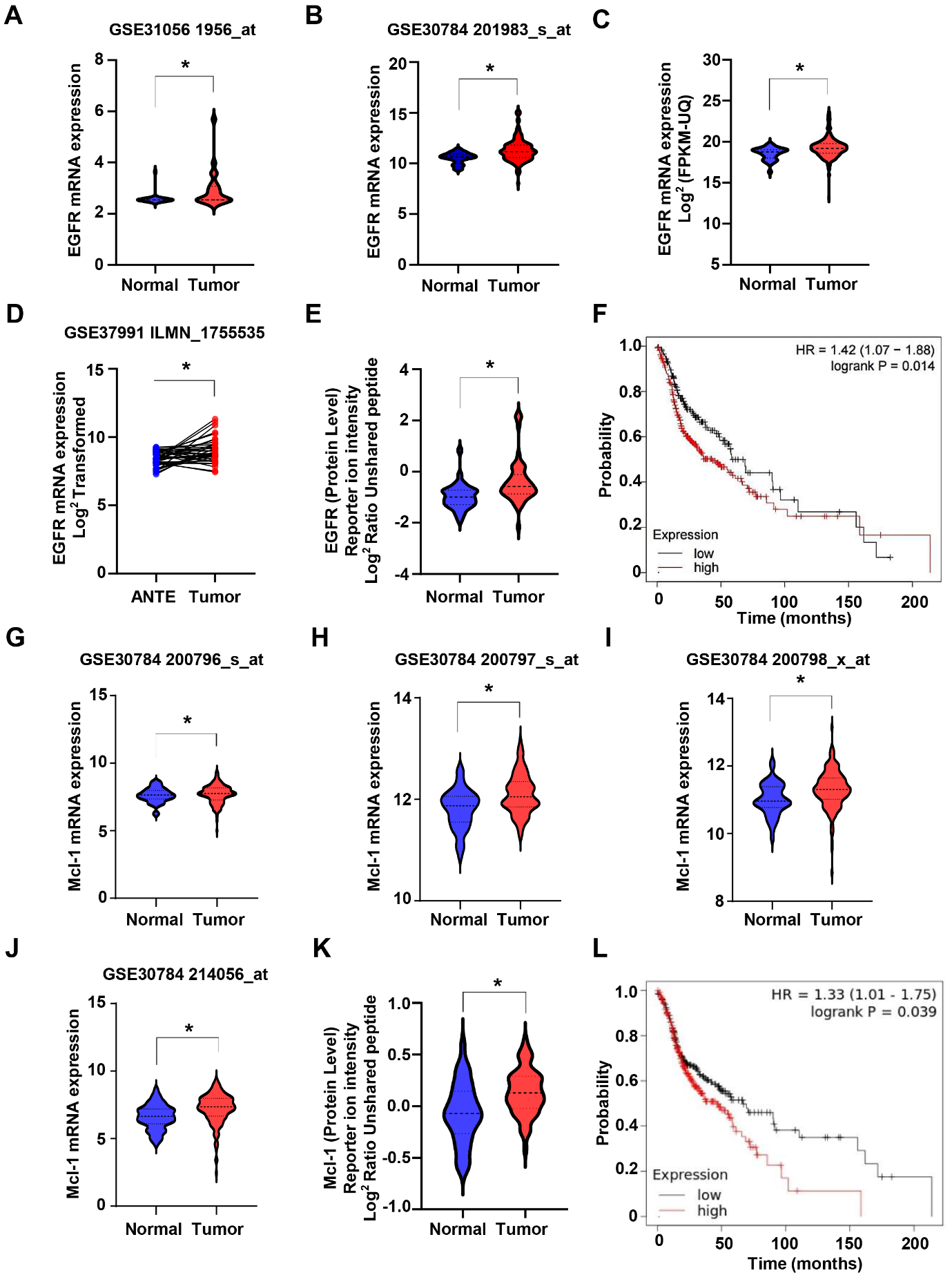
Additional File 1. Correlation between the expression levels of either EGFR or Mcl-1 and prognosis in human HNC.** Comparison of EGFR mRNA expression levels between HNC and normal tissues using GEO (A, B) and TCGA (C) datasets. (D) Analysis of EGFR mRNA expression levels in HNC and adjacent normal epithelial tissues using the GEO dataset. (E) Evaluation of EGFR protein expression levels in HNC and normal tissues using the CPTAC database. (F) Evaluation of the association between EGFR protein expression levels and poor overall survival in HNC patients using the KM plotter analysis. (G–J) Comparison of the Mcl-1 mRNA expression levels between the HNC and normal tissues using the GEO datasets. (K) Analysis of Mcl-1 protein expression levels in the HNC and normal tissues using the CPTAC database. (L) Evaluation of the association between the Mcl-1 protein expression levels and poor overall survival in HNC patients using the KM plotter analysis.


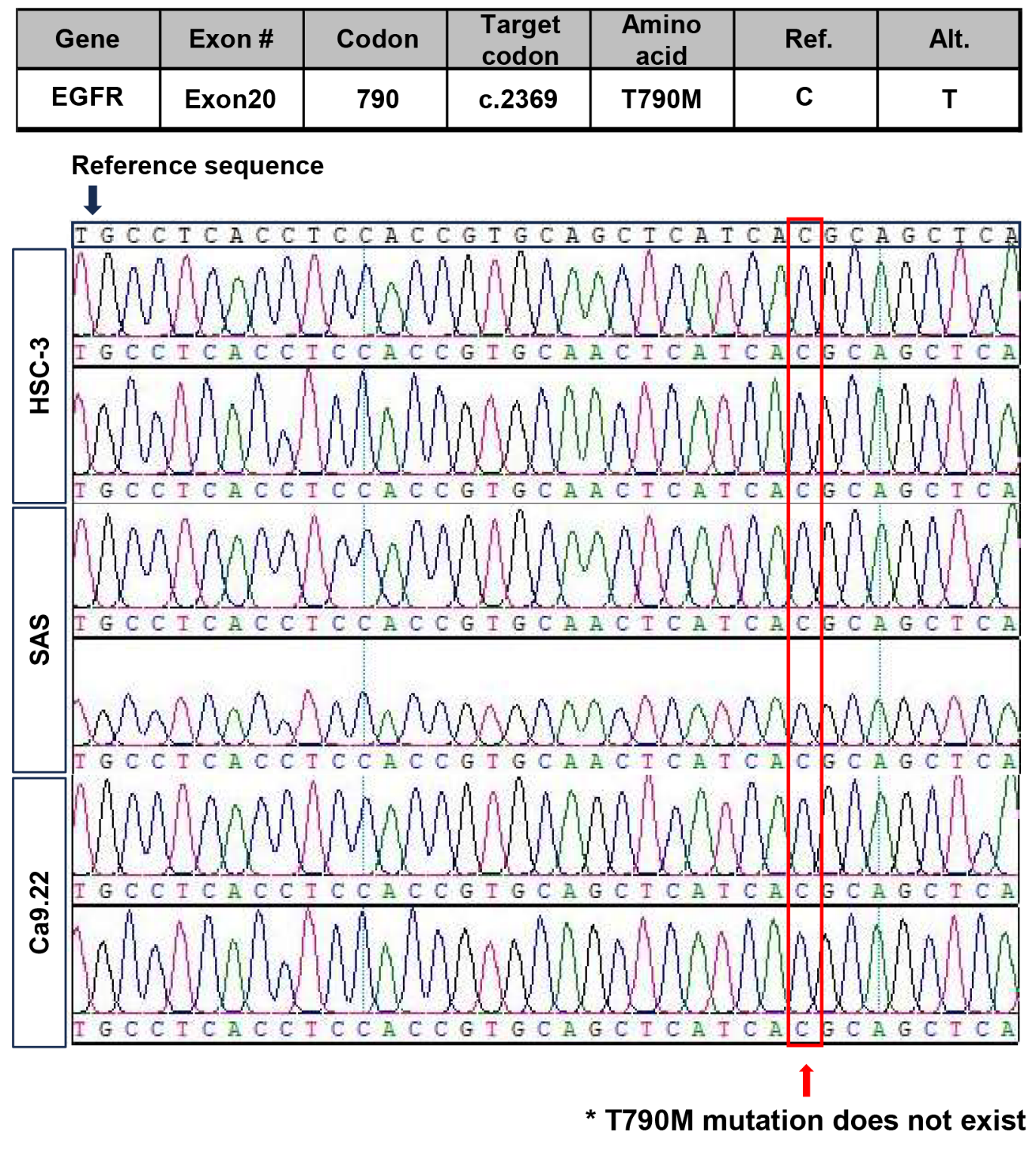


**Additional File 2. Presence of T790M mutation in human oral cancer cell lines.** T790M mutation in exon 20 of the EGFR in three oral cancer cell lines was analyzed by PCR amplification and Sanger sequencing.
